# Process Evaluation of a Community-Based Microbial Larviciding Intervention for Malaria Control in Rural Tanzania

**DOI:** 10.3390/ijerph17197309

**Published:** 2020-10-07

**Authors:** Nina Berlin Rubin, Leonard E.G. Mboera, Adriane Lesser, Marie Lynn Miranda, Randall Kramer

**Affiliations:** 1 Department of Earth System Science, Stanford University, Stanford, CA 94305, USA; 2 SACIDS Foundation for One Health, Sokoine University of Agriculture, 3297 Morogoro, Tanzania; lmboera@gmail.com; 3 Duke Global Health Institute, Duke University, Durham, NC 27710, USA; adriane.lesser@gmail.com (A.L.); kramer@duke.edu (R.K.); 4 Department of Applied and Computational Mathematics and Statistics, University of Notre Dame, Notre Dame, IN 46556, USA; mlm@nd.edu

**Keywords:** process evaluation, malaria, vector control, larval source management, implementation assessment, microbial larvicide, Tanzania

## Abstract

Microbial larviciding can be an effective component of integrated vector management malaria control schemes, although it is not commonly implemented. Moreover, quality control and evaluation of intervention activities are essential to evaluate the potential of community-based larviciding interventions. We conducted a process evaluation of a larval source management intervention in rural Tanzania where local staff were employed to apply microbial larvicide to mosquito breeding habitats with the aim of long-term reductions in malaria transmission. We developed a logic model to guide the process evaluation and then established quantitative indicators to measure intervention success. Quantitative analysis of intervention reach, exposure, and fidelity was performed to assess larvicide application, and interviews with larviciding staff were reviewed to provide context to quantitative results. Results indicate that the intervention was successful in terms of reach, as staff applied microbial larvicide at 80% of identified mosquito breeding habitats. However, the dosage of larvicide applied was sufficient to ensure larval elimination at only 26% of sites, which does not meet the standard set for intervention fidelity. We propose that insufficient training and protocol adaptation, environment and resource issues, and human error contributed to low larvicide application rates. This demonstrates how several small, context-specific details in sum can result in meaningful differences between intervention blueprint and execution. These findings may serve the design of other larval source management interventions by demonstrating the value of additional training, supervision, and measurement and evaluation of protocol adherence.

## 1. Introduction

Of the more than 200 million cases of malaria occurring annually worldwide, 90% take place in sub-Saharan Africa, and 5% of global malaria deaths in 2018 were concentrated in the United Republic of Tanzania [1]. Due to structural constraints within communities and the biological interactions between parasite, vector, and environment, it is difficult to determine best practices for malaria control [2]. Popular malaria reduction strategies, such as deployment of insecticide-treated mosquito nets (ITN) and indoor residual spraying, often result in unintended downstream outcomes such as mosquito avoidance behaviors and vector resistance [3,4]. Larval source management (LSM), though an effective component of integrated vector management (IVM) [5], is largely neglected in malaria control schemes [6]. Encompassing habitat modification, habitat manipulation, biological control, and larviciding, LSM is recommended as a supplementary method for malaria control when breeding sites are “few, fixed and findable” [7] (p. 1). Given concerns about antimalarial drug and insecticide resistance, further progress on the global campaign against malaria requires a wider, more diverse set of approaches, including, potentially, larval source management. 

Larval source management interventions may utilize biological control agents, such as *Bacillus thuringiensis* var. *israelensis* (Bti). This bacterium produces toxins that have proven effective in suppressing larval and pupae production when sufficiently applied to larval breeding habitats [8,9]. Unlike synthetic pesticides, bacterial-origin larvicides do not have negative side effects on human health [10]. In addition, Bti is target species-specific, inexpensive, and environmentally safe; has a low probability of developing resistance; does not require human behavioral change; and does not result in vector avoidance behaviors [3,6,10,11,12,13]. A study in The Gambia showed that Bti application in large sites (i.e., rice paddies) resulted in “consistent suppression of larval development” after treatment with the minimum dosage, and application in small sites (i.e., open pits, puddles) killed all *Anopheles gambiae* larvae within 48 h [8] (p. 7). Combined with ITN use, one group of researchers found that use of *Bacillus*-based larvicides resulted in a twofold reduction in new malaria infections compared to exclusive ITN use [12]. Findings demonstrating a significant decrease in mosquito colonization in man-made aquatic sites also suggest that LSM could be appropriate for urban populations with seasonal larvae populations when incorporated with other malaria control measures [6]. Furthermore, given the relative ease with which the larvicide can be applied, this methodology may be well-suited for community-based programs [6]. Given the apparent benefits of Bti application, it is important to evaluate the feasibility of utilizing this approach to determine what role larval source management should play in integrated vector management. 

For public health interventions, the extent to which activities are implemented according to protocol is critical to the intervention’s efficacy. Program evaluation collects and analyzes information about a project’s efficacy in order to determine whether a program is valuable, cost-efficient, and better than alternatives at producing intended outcomes. Process evaluation is a component of program evaluation that assesses a program’s implementation rather than focusing on its impacts [14]. To determine strengths and weaknesses of programs and their underlying theory and aid in the design of future interventions, process evaluations compare intervention protocol to its real-world actualization [15]. Examining whether elements of programs were delivered according to or deviating from the plan can help explain study results and help differentiate between theory failure and implementation failure [14,15]. If implementation differs greatly from the intended plan, a lack of effect may be explained by implementation error [16]. However, if no significant difference is detected between the original plan and its implementation, there may be an underlying theoretical error if objectives were not realized. Avoiding type III errors–i.e., differences between protocol and implementation–ensures that conclusions regarding an intervention’s effectiveness are accurate [14]. 

Process evaluation of a malarial larviciding intervention enables a distinction to be made between failure to successfully implement a larvicide application program and failure of a successfully implemented larvicide intervention to reduce malaria prevalence in the human population. This study uses the process evaluation approach to examine the implementation of a larviciding intervention for malaria control in rural Tanzania. Given the complexity of context-specific factors that contributed to implementation of this intervention, this paper focuses on the larviciding activities performed by community health workers across 12 villages in Tanzania. This evaluation consists of three components: development of a logic model to identify intervention protocol, quantitative analysis of larviciding activities, and review of interviews with larviciding staff. The focus on assessing the larviciding teams’ ability to apply the correct amount of larvicide enables us to determine whether the program was implemented appropriately, informing future integrated vector management programs. 

## 2. Materials and Methods 

### 2.1. Study Setting

**Figure 1:**
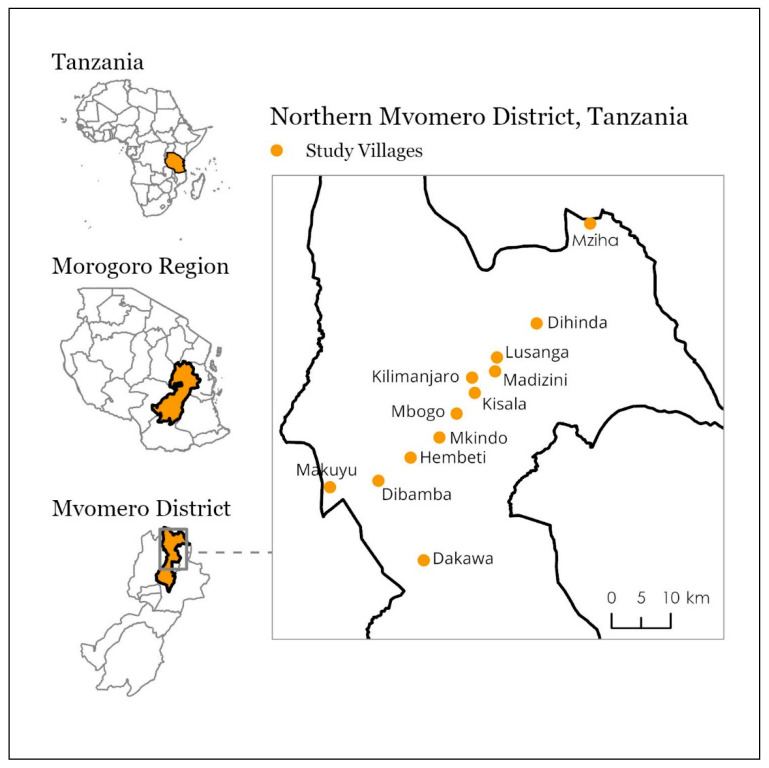
Location of 12 villages included in the larviciding intervention in the Mvomero District of Tanzania. Locations are approximate.

In order to discern the best strategies to combat malaria and reduce its burden, the Mvomero Project in rural Tanzania, named for its location in the Mvomero District, used an implementation science approach by training and deploying community health workers (CHWs) to perform rapid diagnostic testing and treatment for malaria in households (see Kramer et al., 2014 [2]). As an additional component of the Mvomero project, teams of 2 community members (hereafter, larviciding staff) in each village were trained to apply the microbial larvicide *Bacillus thuringiensis* var. *israelensis* (Bti) to potential mosquito breeding habitats to suppress mosquito populations [2]. The overall intervention was conducted across 24 randomly selected villages and split into 4 arms, with 6 villages receiving the following intervention activities: control, rapid testing and treatment, larviciding, and both larviciding and testing/treatment [2]. Larviciding took place during the rainy season of 2012 and 2013 (February–May and January–May, respectively) [5]. This process evaluation focused only on the 12 villages included in the larviciding intervention (see Figure 1). In each of these 12 villages, 2 community members were recruited by local leaders and trained by entomologists and a larvicide specialist to both identify mosquito species and apply an appropriate amount of larvicide to the site [2,5]. Following the identification of larval habitats—such as ponds, rice paddies, brick pits, and temporary sites from rainfall—larviciding staff recorded larvae and pupae counts and habitat size, then applied microbial larvicide at a dosage specific to the biological control agent Bti: 5 kg per hectare. Larviciding staff were instructed to revisit sites every 7 to 10 days. Trained entomologists served as supervisors who were instructed to visit each of the villages roughly every 2 weeks to conduct quality control checks and restock supplies when necessary [2]. 

**Figure 2:**
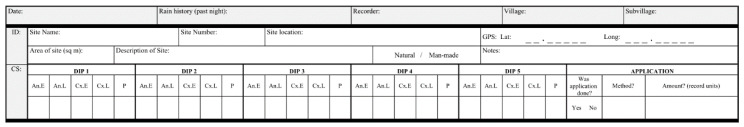
Site form filled out by larviciding staff at each larval breeding site identified.

After larviciding staff identified a breeding habitat, they were instructed to dip a 350 mL mosquito dipper at 1 to 5 locations across the site. Larviciding teams were given reference materials (in Kiswahili) to refer to at each site to determine site area, the corresponding amount of larvicide necessary to eliminate larval populations at the site, and the matching number of handfuls or cups to apply. When larvae or pupae were present, larviciding staff were instructed to apply the manufacturer recommended level of 5 kg per hectare of larvicide by hand for small sites or via a sprayer machine for large sites. To document their efforts, staff were instructed to fill out a larviciding site form (see Figure 2, translated to English) at each site visit. The form included pertinent information such as site location (GPS coordinates), site description, the designation of natural versus manmade, the number of larvae/pupae found for each dip (divided into *Anopheles* early and late instar, *Culex* early and late, and pupae), site size (in square meters), and larvicide application method and amount (in various units). Data from the larviciding forms are available upon request from the authors.

### 2.2. Logic Model

**Figure 3:**
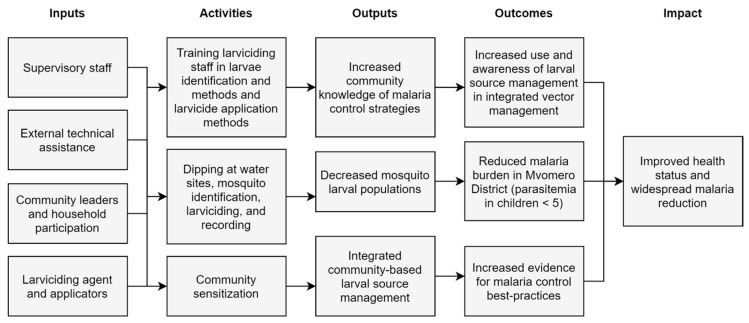
Logic model for the malarial larviciding component of the Mvomero Project intervention.

Logic models, also known as program theories, visually depict the underlying rationale driving an intervention [17]. These systematic displays help determine structural and financial needs, link inputs to products, and communicate theory to potential investors [17,18]. A logic model, as the first step in this process evaluation, was developed (Figure 3) to detail the theory and components of the Mvomero Project larviciding intervention. The Mvomero Project was designed to test if vector management and reduction subsequently result in parasitic reduction and reduced malarial burden. Creation of the logic model enabled post-hoc analysis of the consistency between the intervention’s blueprint and the intervention’s implementation [19]. The ensuing process evaluation examines the realization of inputs and activities detailed in the logic model, primarily supervisory staff, larviciding staff training, larvicide application, and activity recording. Then, inferences can be made with respect to how intervention implementation may have contributed to the resulting outputs, and thus how discrepancies between the logic model and the actualized larviciding intervention could result in outcomes other than those anticipated. This visualization aids recognition of intervention assumptions and where links may have been expected and not realized, and, following evaluation, helps guide intervention refinement [16]. 

### 2.3. Data Analysis

Although implementation evaluation can have multiple objectives according to the priorities of stakeholders involved [14], we follow the approach outlined by Steckler and Linnan (2002) [14]. In this study, we focus on reach, exposure, fidelity, and resources. Three quantitative indicators shown in Table 1 were developed to reflect these components to assess the intervention’s implementation as a whole and in each of the 12 villages. Indicators were established using data compiled from the larviciding sheet forms filled out by larviciding staff. Data cleaning was performed to correct erroneous errors in site size and larvicide application amounts when context was available, as well as to convert larvicide application amounts to kilograms. 

#### 2.3.1. Quantitative Indicators

The percentage of all sites visited that received larvicide, both with and without larval presence reported, was calculated as a measure of intervention *reach*. This was calculated by dividing the number of breeding habitats where larvicide was applied by the total number of breeding sites visited. To analyze *exposure,* we calculated the average and median dosage of larvicide applied to each breeding site for each village and across the intervention. For each site, we divided the amount of larvicide applied in kilograms by the size of the breeding habitat in hectares to determine dosage in kilograms per hectare. The average and median dosage was calculated at the village level and the average and median dosage of larvicide applied at each breeding site was computed across all breeding habitats. Lastly, the percentage of breeding habitats receiving an adequate dosage (as defined by the manufacturer as ≥ 5 kg per hectare) of larvicide per village and overall were computed to assess intervention *fidelity*. 

In advance of analysis, the authors determined standards for distinguishing between component success and failure as per process evaluation best practices [14]. Success on the quantitative indicators was defined as

- Greater than 75% of breeding habitats receiving larvicide application (*reach*);
- At least 5 kg per hectare of larvicide being applied at each site (*exposure*);
- At least 50% of sites receiving an adequate dosage according to manufacturer instructions (*fidelity*).

Data from the second year of the intervention (2013) were selected for this evaluation.

#### 2.3.2. Qualitative Indicators

Other components of the intervention were considered qualitatively, given the variety of experiences and settings across the 12 villages included in the intervention. Qualitative analysis of in-depth interviews conducted with larviciding staff in 2 villages and supervisor reports was performed to provide additional context to quantitative results. 

## 3. Results

### 3.1. Quantitative Analysis

#### 3.1.1. Overall Larviciding Activity

Across the 12 villages, larviciding staff reported visiting 4498 sites identified as potential mosquito breeding habitats during the study period. Of all sites visited, larviciding staff recorded that 3007 (67%) had mosquito larvae or pupae presence. 

#### 3.1.2. Reach: Percentage of Visits with Larvicide Application

**Figure 4:**
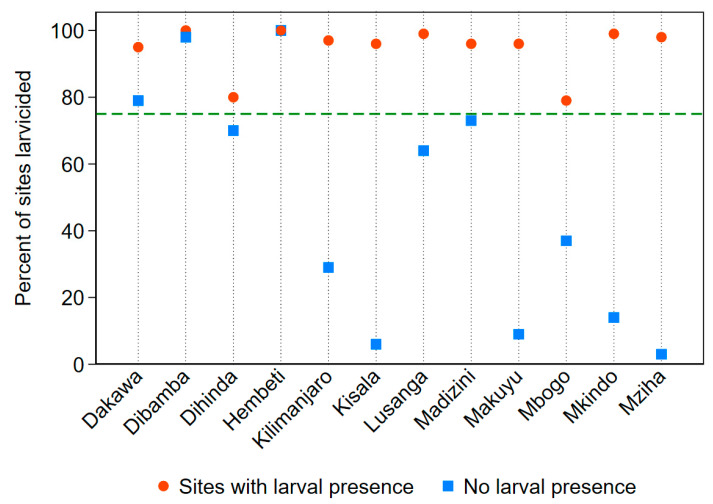
Percentage of sites visited receiving larvicide application of any amount for sites with and without reported larval presence. The green dashed line depicts the success standard of 75%.

Larvicide was applied at 3636 of the 4498 sites visited (81%). At the village level, the percentage of all sites visited that were larvicided ranged from 27% (Makuyu) to 100% (Hembeti). Of the 3007 visits where there was larval presence reported, staff applied larvicide at 2840 of them (94%). Village by village frequencies of larvicide application when larvae or pupae were reported as present ranged from 79% (Mbogo) to 100% (Dibamba and Hembeti). For the 1491 site visits that did not report larval presence, 761 received larvicide (51%). Village percentages for sites without larval presence receiving larvicide ranged from 3% (Mziha) to 100% in Hembeti. Figure 4 displays the percentage of site visits where larvicide was applied for sites with and without larval presence in comparison to the 75% success standard established prior to data analysis.

#### 3.1.3. Exposure: Mean and Median Larvicide Dosage Applied

**Figure 5:**
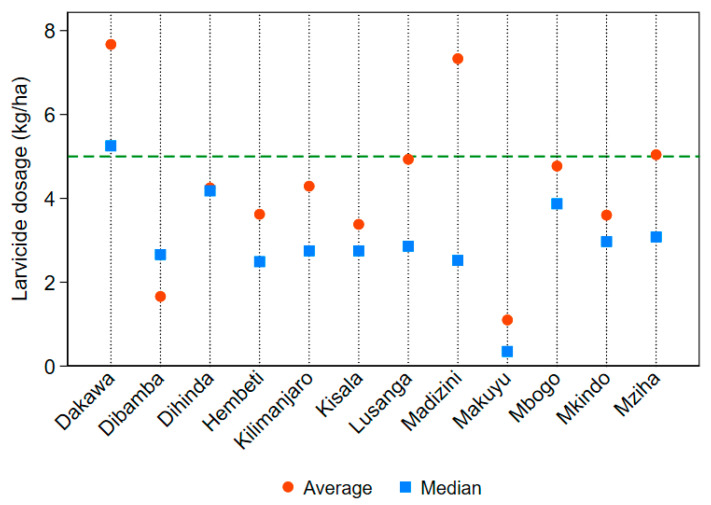
Average and median larvicide application rates (in kilograms per hectare) by village. The green dashed line depicts the standard of 5 kg/ha.

Adequate application was defined as applying at least 5 kg per hectare of larvicide material per visit as per staff instruction and manufacturer requirements [20]. Mean and median larvicide application dosage by village, alongside the instructed success standard of 5 kg per hectare, is displayed in Figure 5. The mean larvicide application rate across the 12 villages was 4.70 kg per hectare. The average dosage of larvicide applied per village ranged from 1.10 kg/ha in Makuyu to 7.67 kg/ha in Dakawa. The median larvicide application dosage across the intervention was 2.87 kg per hectare. The difference between the average and the median dosages suggests that the distribution of larviciding dosage was positively skewed, and over-application of larvicide occurred at some habitats. Whereas sites at 4 out of 12 villages met or exceeded the instructed dosage using the mean statistic, only one village did so when using the median. The latter metric is more appropriate given evidence of outliers in larvicide application. 

#### 3.1.4. Fidelity: Percentage of Sites Receiving an Adequate Application Dosage

**Figure 6:**
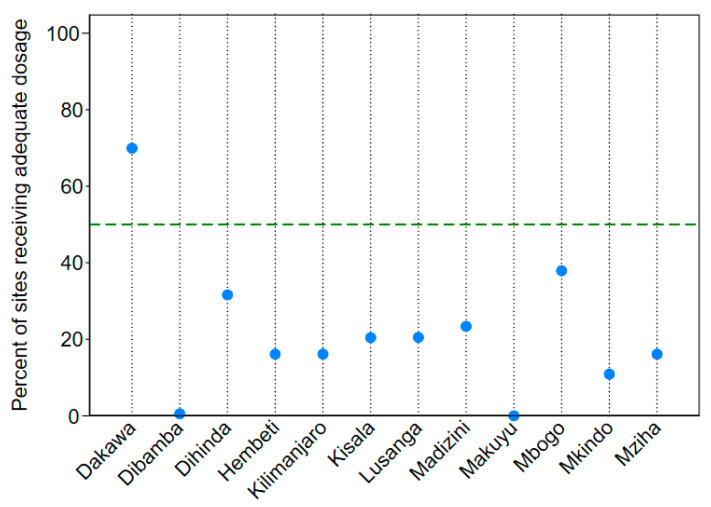
Percentage of sites receiving larvicide that were larvicided with an adequate dosage of at least 5 kg per hectare, by village. The green dashed line depicts the standard of 50% of sites receiving an adequate dosage of larvicide application.

Of all the sites receiving any amount of larvicide in 2013, 26% (936 out of 3636 sites) received an adequate dosage of at least 5 kg per hectare. Village by village adequate application percentages (shown in Figure 6) ranged from 0% in Makuyu and Dibamba to 70% in Dakawa. The frequency of adequate application for sites larvicided with larval presence reported was 25%. Given the small difference between the overall adequate larviciding frequency and the adequate larviciding frequency when larvae were reported as present, precautionary larviciding when larvae were not present likely did not negatively skew overall adequate larviciding frequency. 

#### 3.1.5. Additional Analysis of Application Adequacy 

Given the results above suggesting low levels of adequate larvicide application, we performed additional analysis to determine factors influencing larviciding dosage, including site size and application method. Analysis of larvicide application adequacy (≥5 kg/ha) across different breeding habitat sizes revealed that the frequency of adequate application of larvicide increased significantly for sites over 0.035 hectares, particularly for sites between a quarter and a half of a hectare large (approximately the largest 20% of sites). Breeding habitats of different sizes necessitated different larvicide application techniques—larger sites were typically treated using machine sprayers operated by the larviciding staff, whereas smaller sites received treatment via hand application. Larval habitats treated by machine sprayers received adequate dosages of larvicide at 43% of visits, whereas habitats treated with larvicide by hand received an adequate dosage at only 15% of visits, a nearly 30% difference.

### 3.2. Qualitative Analysis

In-depth interviews were conducted with larviciding staff in 2 of the 12 villages where the larviciding intervention took place. Emerging themes from the interviews with larviciding staff suggest that there was a need for greater communication with supervisory staff. Larviciding staff reported that they believed the number of mosquito larvae had decreased since the start of the intervention. Additionally, larviciding staff expressed confidence in their larviciding abilities, and believed their work was manageable though dependent on issues with rain and their larviciding partner. Finally, staff suggested that a lack of reference sheets—which the job aids used on site to determine the amount of larvicide necessary for a site of a particular size—may have caused issues in instance when they had forgotten their training. 

## 4. Discussion

Larval presence clearly triggered action by larviciding staff, as evidenced by overall larviciding frequency across the study villages, suggesting the intervention was successful on the *reach* indicator of the process evaluation. In terms of the *exposure* component of evaluation, the difference in larviciding frequency between sites with and without larval presence demonstrates that staff largely responded appropriately to larval presence and administered larvicide when necessary. However, results suggest that some staff may have been applying larvicide to all sites on a precautionary basis, irrespective of larval presence. Over-larviciding reduces overall efficiency in terms of resource and time management. The larviciding intervention failed to meet standards set along the *fidelity* component of this process evaluation, as an insufficient dosage of larvicide was applied at most breeding sites. This is problematic given that inadequate larvicide application may have consequences for malaria vector populations and downstream transmission. We also found that larvicide dosage was associated with larvicide application method, as larger sizes more frequently received a higher dose where machine sprayers were used to disperse larvicide. We posit that this association is in part due to the difficulty of estimating the appropriate larvicide amount to apply when using a non-standard size cup or mug for measurement, as was the case when staff applied larvicide by hand for small sites. 

Given quantitative findings and qualitative assessment/context provided by intervention administrators and staff, we propose three factors that contributed to low intervention *fidelity*: human error, insufficient training and protocol modification, and environment and resource issues. Data management and cleaning in advance of and during this process evaluation uncovered recording errors in forms filled out by larviciding staff—ranging from evidently incorrect site size estimation to incorrect larvicide unit notation—highlighting how individual-level human error can affect intervention-level outcomes. Further, training sessions may not have sufficiently prepared larviciding staff for the breadth of work they were expected to complete. Job aids (such as site size to larvicide amount conversion sheets) may have been too difficult to utilize at each site visit and filling out the site form posed a significant burden for the staff. Finally, issues with sprayer pumps and transportation during rainy periods may have limited larviciding abilities. These small factors, in sum, likely contributed to differences between the intervention blueprint and its eventual implementation. 

### Recommendations to Improve Future Interventions

Other evaluations of malarial larviciding interventions have similarly found that these programs are highly complex and context-specific, requiring acute attention to detail. For large-scale larviciding programs, coordination with Ministries of Health, local community leaders, and research groups is essential to achieve widespread success in rolling out activities [21]. Although this requires extensive planning and arrangement in advance, the day-to-day activities of larviciding teams and supervisors must be somewhat flexible to account for unanticipated changes such as weather events or issues with machine sprayers [21,22]. The need for close supervision of larviciding staff to ensure quality control of the intervention, coupled with budgetary concerns and other factors, demonstrates the high level of complexity involved in implementing these programs. However, evaluation of these complexities is lacking. Measuring and reporting on the gap between intervention protocol and implementation enables researchers and administrators to better understand intervention delivery and helps them discover unforeseen factors limiting or contributing to achievement of short- and long-term program impacts. For example, for hand application of smaller sites, larviciding staff should be provided with a measured cup inscribed with larvicide corn granule amounts (in grams or in site size). Using a measuring cup would increase the accuracy of larviciding staff as they would not have to estimate fractions of handfuls and gram amounts in their hand, and it would provide a standard form of measurement across larvicide spray teams. It may also negate the need to refer to complex job aids. These efforts should occur throughout the intervention process—as issues commonly identified after the fact are unpredictable and should be addressed fluidly—and post-hoc [22].

It is important to consider the geographic and environmental conditions that foster larval population growth when determining locations that might be appropriately targeted by larviciding interventions. For example, heavy rainfall was common in Dakawa and Mkindo, resulting in the proliferation of large potential breeding grounds in these villages, while other villages remained dry for some periods during the intervention. Given the difficulty in ensuring an adequate amount of larvicide is applied to breeding habitats in order to reduce larval populations capable of transmitting malaria, larviciding activities could focus less on temporary sites and more on continually productive, long-term larval habitats. Targeting dry season habitats, which are present throughout the year and are continually productive sources of larval abundance, could be made a higher priority in further larval source management efforts. Although it is necessary to consider every possible breeding site as posing a risk of malaria transmission, this strategy may increase intervention efficacy, and reducing vector populations during the dry season could also reduce population growth during the rainy season [23]. This would be both more manageable for larviciding staff—reducing the number of breeding sites they visit—as well as for supervisors and intervention administrators, while potentially achieving comparable or more tangible impacts. If larval populations are receiving adequate dosages of larvicide more frequently, there is a higher likelihood that they will be unable to proliferate and pose the threat of malarial transmission to communities in the area. In addition, intervention administrators could consider adjusting the timing of larviciding activities. Larviciding earlier in the rainy season might reduce mosquito populations such that mosquito prevalence would be limited later in the season.

## 5. Conclusions

This paper has shown that the implementation of larviciding activities in the Mvomero Project differed from protocol in several important ways. Although larvicide application was widespread and frequent, application dosage was inappropriately low, and the vast majority of sites received less larvicide than necessary to optimally eliminate larval populations. Larvicide was found to be adequately applied less frequently when applied by hand than by machine. Further training of larviciding staff for hand application or increased use of machine sprayers for small sites is necessary to increase the adequacy of larvicide dosing. Furthermore, more cadenced supervision and monitoring of larviciding teams’ activities may decrease the disjuncture between intervention protocol and implementation. Future larviciding interventions may benefit from this evaluation, which underscores the importance of training, supervision, and intervention adaptation to local resource or environmental constraints. Recommendations proposed here should be considered for future LSM interventions, which may be of increasing importance due to concerns with other malarial control strategies. More generally, this process evaluation emphasizes the importance of measuring and reporting on protocol adherence in order to meaningfully measure and interpret intervention impact.

## Figures and Tables

**Table 1:** Indicators and analysis methods.

Indicator	Analysis	Metrics	Standard
Reach	Quantitative	Percentage of breeding habitats identified with subsequent larvicide application	>75%
Exposure	Quantitative	Mean and median larvicide application dosage	≥5 kg per hectare
Fidelity	Quantitative	Percentage of breeding habitats identified receiving an adequate dosage of larvicide (≥5 kg/ha)	≥50%
Resources	Qualitative	Review of supervisor reports and in-depth interviews with larviciding staff	Not applicable
